# Lateral Transmission of Yeast Symbionts Among Lucanid Beetle Taxa

**DOI:** 10.3389/fmicb.2021.794904

**Published:** 2021-12-14

**Authors:** Gaku Ueki, Sheng-Nan Zhang, Xue-Jiao Zhu, Xiu-Jun Wen, Koji Tojo, Kôhei Kubota

**Affiliations:** ^1^Department of Biology, Graduate Faculty of Science, Shinshu University, Matsumoto, Japan; ^2^Laboratory of Forest Zoology, Department of Forest Science, Graduate School of Agricultural and Life Sciences, The University of Tokyo, Bunkyo, Japan; ^3^Guandong Key Laboratory for Innovative Development and Utilization of Forest Plant Germplasm, College of Forestry and Landscape Architecture, South China Agricultural University, Guangzhou, China

**Keywords:** phylogenetic analysis, species distribution model, ecological space, *Scheffersomyces*, *Prismognathus dauricus*, *Prismognathus angularis*, *Platycerus viridicuprus*, *Platycerus hongwonpyoi*

## Abstract

To deepen understanding the evolutionary process of lucanid–yeast association, the lateral transmission process of yeast symbionts among stag beetle genera *Platycerus* and *Prismognathus* around the border between Japan and South Korea was estimated based on molecular analyses and species distribution modelings. Phylogenetic analyses were based on yeast ITS and IGS sequences and beetle *COI* sequences using *Prismognathus dauricus* from the Tsushima Islands and *Pr. angularis* from Kyushu, Japan, as well as other sequence data from our previous studies. The range overlap based on the species distribution model (SDM) and differentiation in ecological space were analyzed. Based on the IGS sequences, Clade II yeast symbionts were shared by *Platycerus hongwonpyoi* and *Pr. dauricus* in South Korea and the Tsushima Islands, and *Platycerus viridicuprus* in Japan. Clade III yeasts were shared by *Pr. dauricus* from the Tsushima Islands and *Pr. angularis* in Japan. During the Last Interglacial period when the land bridge between Japan and the Korean Peninsula existed, range overlap was predicted to occur between *Pl. viridicuprus* and *Pr. dauricus* in Kyushu and between *Pr. dauricus* and *Pr. angularis* in Kyushu and the Tsushima Islands. The ecological space of *Pl. hongwonpyoi* was differentiated from that of *Pl. viridicuprus* and *Pr. angularis*. We demonstrated the paleogeographical lateral transmission process of *Scheffersomyces* yeast symbionts among lucanid genera and species: putative transmission of yeasts from *Pr. dauricus* to *Pl. viridicuprus* in Kyushu and from *Pr. angularis* to *Pr. dauricus* in Kyushu or the Tsushima Islands. We also found that the yeast symbionts are likely being replaced in *Pr. dauricus* on the Tsushima Islands. We present novel estimates of the lateral transmission process of microbial symbionts based on phylogenetic, SDM and environmental analyses among lucanid beetles.

## Introduction

In studies of insect–fungus symbiotic associations, particular attention has been paid to the mode of fungal transmission, because it is deeply related to the dependency between partners and the evolution of symbiotic systems. Vertical transmission (parent to offspring) is common in insect–fungus mutualism, whereas lateral transmission may occur between neighboring nests (Biedermann and Vega, 2020). Generally, the presence of fungus-carrying organs in insects indicates obligate dependencies and fungus productivity emphasizes genetic homogeneity (ants: Mueller et al., 1998; termites: Aanen et al., 2009). Meanwhile, lateral fungal transmission increases diversity and rarely results in obligate dependency evolution in insect-fungus symbiotic systems (West et al., 2015; Biedermann and Vega, 2020).

Stag beetles belonging to the family Lucanidae feed on decaying wood during the larval stage (Tanahashi et al., 2010, 2018; Tanahashi and Kubota, 2013). Adult lucanid females commonly possess a mycangium, an exoskeletal organ on the dorsal side of the abdominal tip carrying symbiotic microorganisms (Tanahashi et al., 2010; Tanahashi and Hawes, 2016; Kubota et al., 2020). The yeasts belong to the genus *Scheffersomyces* Kurtzman and Suzuki, 2010, a xylose-fermenting yeast group (Du Preez and Prior, 1985; Jeffries and Kurtzman, 1994; Olsson and Hahn-Hägerdal, 1996; Jeffries et al., 2007) including *S*. *stipitis* (Pignal) Kurtzman and Suzuki, 2010, *S*. *segobiensis* (Santa María and C. García) Kurtzman and Suzuki, 2010 or closely related species (Tanahashi et al., 2010; Kubota et al., 2020). It is nearly the only fungus found in almost all female mycangia of *Platycerus* Geoffroy, 1762; *Prismognathus* Motschulsky, 1860; *Dorcus* MacLeay, 1819; *Lucanus* Scopoli, 1763; and other lucanid species that feed on white rot wood (Tanahashi et al., 2010, 2017; Kubota et al., 2020). Although these *Scheffersomyces* yeast symbionts appear to serve an important role for the host stag beetles, their exact symbiont function remains unknown (Kubota et al., 2020).

Kubota et al. (2020) documented the co-evolutionary relationship between host lucanid beetles and their yeast symbionts based on a phylogeographic analysis of *Platycerus*. All yeast colonies from individual females appear to exhibit little to no genetic variation, and colonies from a particular lucanid population are closely related to one other (Tanahashi et al., 2017; Kubota et al., 2020). However, the symbiont and host beetle phylogenies are not at all congruent with each other, suggesting the occurrence of lateral transmissions and replacements of yeast symbionts between lucanid populations or species. Although the *Scheffersomyces* yeasts are temporarily found in the larval gut of wood-feeding beetles of the genera of Passalidae, Cerambycidae, Scarabaeidae, and Buprestidae (Santa-María and García-Aser, 1977; Suh et al., 2003, 2005), *Scheffersomyces* are not easily found in decaying wood except for larval galleries of lucanid species based on our observations and Roets and Oberlander (2020). Meanwhile, *Scheffersomyces* species, including *S. stipitis*, *S. segobiensis*, and their relatives, are almost always found in mycangium and gut of the many lucanid taxa (e.g., *Platycerus*, *Prismognathus*, *Dorcus*, and *Lucanus*, which are common in East Asia). Therefore, this group of *Scheffersomyces* appears to have essentially diverged along with lucanid beetles (Kubota et al., 2020).

**FIGURE 1:**
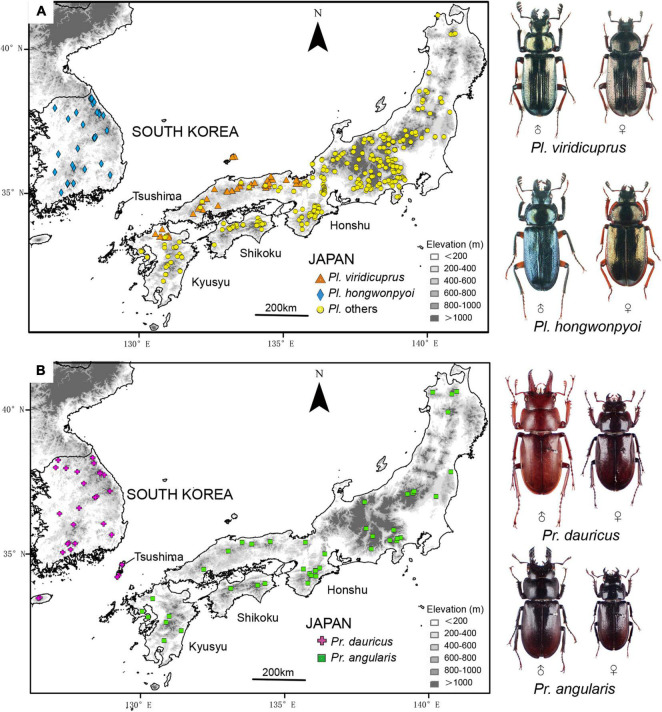
Distribution and occurrence data in Japan and South Korea for **(A)** *Platycerus viridicuprus* and *Pl. hongwonpyoi*; and **(B)** *Prismognathus dauricus* and *Pr. angularis*. Background map shows elevation, where higher intensity of the gray scale represents higher altitude.

Here, we focus on the yeast symbionts of *Platycerus* and *Prismognathus* lucanid species in Japan and South Korea. For *Platycerus*, only one species, *Platycerus hongwonpyoi* Imura and Choe, 1989, is known in Korea, whereas 10 species forming a monophyletic clade are recognized in Japan (Imura, 2010; Kubota et al., 2011). For *Prismognathus*, one species, *Prismognathus dauricus* (Motschulsky, 1860) occurs in Korea, and three species, *Prismognathus angularis* Waterhouse, 1874; *Pr. tokui* Kurosawa, 1975; and *Pr. dauricus* are found in Japan. *Prismognathus tokui*, which is endemic to Yakushima Island, is often treated as a subspecies of *Pr. angularis* (Fujita, 2010). In Japan, *Pr. dauricus* in Japan only occurs on the Tsushima Islands, located between the Japanese Archipelago and the Korean Peninsula (Figure 1). *Prismognathus angularis* is not found on the Tsushima Islands. These lucanid species are adapted to mature cool temperate deciduous broad-leaved forests, of which some species (e.g., *Pr. dauricus*) are also found in warm temperate evergreen broad-leaved forests.

Tanahashi et al. (2017); Kubota et al. (2020), and Zhu et al. (2020a) analyzed the sequences of the internal transcribed spacer (ITS) region including the initial portion of the 26S ribosomal RNA gene (*26S rRNA*) (about 650 bp), and the inter-genetic spacer (IGS) region including the *26S rRNA*, IGS1, 5S ribosomal RNA gene (*5S rRNA*), IGS2, and the 5S ribosomal RNA gene (*18S rRNA*) (>2,000 bp), using all of the *Platycerus* and *Prismognathus* species, except for Japanese *Pr. tokui*. The ITS sequences of the yeast symbionts of *Platycerus* and *Prismognathus* in Japan and Korea are the same or little varied. Based on the IGS sequences, which are suitable for detecting strain-level genetic differences, the yeast symbionts form three distinct major clades (Tanahashi et al., 2017; Kubota et al., 2020). Of these, Korean *Pl. hongwonpyoi* and *Pr. dauricus*, and most populations of Japanese *Platycerus viridicuprus* Kubota et al., 2008, share an IGS lineage of yeast symbionts, which suggests recent lateral yeast transmission among these species when Japan and Korea were connected by land (Zhu et al., 2020a). This is the only report that phylogenetically distant lucanid taxa (*Platycerus* and *Prismognathus*) share yeasts of the same lineage.

The presence of the mycangium and co-evolutionary relationships in lucanid–yeast symbiotic systems suggest obligate dependency. The symbiotic relationship of *Platycerus* and *Prismognathus* with yeasts around the border of Japan and Korea offers the opportunity to estimate the rare lateral transmission process. *Pr. dauricus* is also distributed within the Tsushima Islands between Japan and Korea, where no *Platycerus* species are found. However, until now, no studies have examined the yeast symbionts of *Pr. dauricus* on the Tsushima Islands.

Species distribution models (SDMs) are statistical models that use observed distributional occurrence data to infer species’ ecological requirements and to map their potential habitat (Austin, 2002). SDM approaches have become a widely applied tool in not only macroecological studies, conservation strategies and the identification of niche evolution, but also in quantifying ecological divergence in closely related species (Zimmermann et al., 2010; Biber et al., 2020; Boys et al., 2021). These SDMs have been paired with paleoclimate data to hindcast past distributions and to understand how they shape current population structures (Forester et al., 2013; Yannic et al., 2014; Costa and Schlupp, 2020). Here, we focus on quantifying the range overlap and niche divergence among *Platycerus* and *Prismognathus* species using the Maximum Entropy Algorithm (MaxEnt). This approach is a high-performing and highly popular method for SDMs (Phillips et al., 2006) and provides the opportunity to explore the transmission process of yeast symbionts among lucanid taxa.

In the present study, to deepen understanding the evolutionary process of lucanid–yeast association, we examined the possible yeast lateral transmission or incomplete lineage sorting following ancestral polymorphism among the stag beetle genera *Platycerus* and *Prismognathus*, using *Pr. dauricus* from the Tsushima Islands and additional samples from a paleogeological perspective. We analyzed the evolutionary relationships among their yeast symbionts based on molecular analyses and predicted the past range and niche overlap among genera based on SDMs. Lastly, we discuss the lateral transmission process of yeast symbionts among species and genera.

## Materials and Methods

### Genetic Analyses

#### Insects

Host insect sample collection sites in this and previous studies are presented in Supplementary Table 1 and Supplementary Figure 1. Isolated yeast strains from this and previous studies are shown in Supplementary Table 2. *Prismognathus dauricus* were obtained as adults at sites 34 and 35 (Supplementary Figures 1, 2). *Prismognathus angularis* were collected as larvae from decaying wood at sites 30 and 31. These larvae were reared individually to adulthood using the same wood that they inhabited in the field. Finally, we obtained seven adult females of all *Prismognathus* species collected from four sites in Japan.

#### Yeast Isolation From Adult Females and DNA Sequences of Yeast Symbionts

The protocols of yeast isolation and determining of yeast DNA sequences were the same as in Kubota et al. (2020). They are described in Supplementary Appendices 1, 2. The primer information is shown in Supplementary Table 3. We used the MAFFT online service (Katoh et al., 2019) to align the sequences and determined 15 ITS and 15 IGS sequences of 56 yeast colonies from five *P. dauricus* and two *P. angularis* females (eight colonies per female) in total.

For the ITS analysis, we aimed to determine the phylogenetic positions of the yeast symbionts of *Platycerus* and *Prismognathus* within those of lucanid species and *Scheffersomyces* species. Thus, we used 46 yeast ITS sequences, of which 38 were symbionts of lucanid beetles reported in previous studies (one from *Pr. dauricus* of South Korea, two from *Pr. angularis*, 14 from Japanese *Platycerus* species, eight from *Pl. hongwonpyoi* of South Korea, and 13 from other lucanid species) (Tanahashi et al., 2017; Kubota et al., 2020; Zhu et al., 2020a; Supplementary Table 2).

For the IGS analysis, we focused on the phylogenies of the yeast symbionts of the four target species within those of *Platycerus* and *Prismognathus* species. We determined 15 IGS sequences from 56 symbiotic yeast isolates from five *Pr. dauricus* females and two *Pr. angularis* females. We also used 73 yeast ITS sequences, of which 72 were symbionts of lucanid beetles reported in previous studies (one from *Pr. dauricus* of South Korea, two from *Pr. angularis*, 61 from Japanese *Platycerus* species, and eight from *Pl. hongwonpyoi* of South Korea) (Tanahashi et al., 2017; Kubota et al., 2020; Zhu et al., 2020a; Supplementary Table 2). The IGS sequences of *S. segobiensis* were used as the outgroup of the yeast symbionts of *Platycerus* and *Prismognathus* species, because the former is closely related to, but distinct from the latter clade (Tanahashi et al., 2017).

#### DNA Sequences of Insect Hosts

Notably, both mitochondria and symbiotic yeasts are essentially transmitted from mother to offspring. Because it is reasonable to compare evolutionary patterns between the cytochrome oxidase subunit I (*COI*) gene of the host beetles and the IGS gene of the yeast symbionts, we sequenced the *COI* gene of the host beetles. Most sequence data for *COI* used in this study were from previous studies, as listed in Supplementary Table 2 (Kubota et al., 2010, 2011, 2020; Kubota and Kubota, 2011; Tanahashi et al., 2017; Zhu et al., 2020a).

The protocol of determining of host beetle *COI* sequences was the same as in Kubota et al. (2020). They are described in Supplementary Appendix 3. The primer information is shown in Supplementary Table 3. To align the sequences, we used the same methods as with the yeast DNA sequences. For the *COI* analysis, we focused on the phylogenetic relationship between *Platycerus* and *Prismognathus* species in Japan and South Korea. We determined seven *COI* sequences (five *Pr. dauricus* females and two *Pr. angularis* females).

#### Phylogenetic Analyses

We constructed a haplotype matrix for each of the ITS and IGS regions of yeasts and the *COI* gene of beetles using the DnaSP v. 6.12.01 software package (Rozas et al., 2017). Aligned haplotype sequences were used to construct phylogenetic trees based on maximum likelihood (ML) and Bayesian inference (BI) methods. ML trees were constructed using RAxML v. 8.2.9 (Stamatakis, 2014) under the best-fit substitution model selected using Kakusan4 (Tanabe, 2007) based on Schwarz’s Bayesian information criterion (BIC; Schwarz, 1978). Confidence at each node was assessed using 1,000 bootstrap replications. BI trees were constructed using MrBayes5D v. 3.2.6 (Tanabe, 2008), a modified version of MrBayes (Huelsenbeck and Ronquist, 2001), under the best-fit substitution model by Kakusan4, for 10 million generations (samplefreq = 1,000), with the first 1,000,000 generations discarded as “burn-in.” The Bayesian log likelihood trace files, burn-in times, and summary statistics of estimated parameters were visualized using Tracer ver. 1.6 (Rambaut and Drummond, 2007). Tree information was visualized and edited using FigTree v. 1.3.1 (Rambaut, 2009).

### Species Distribution Modeling

**FIGURE 2:**
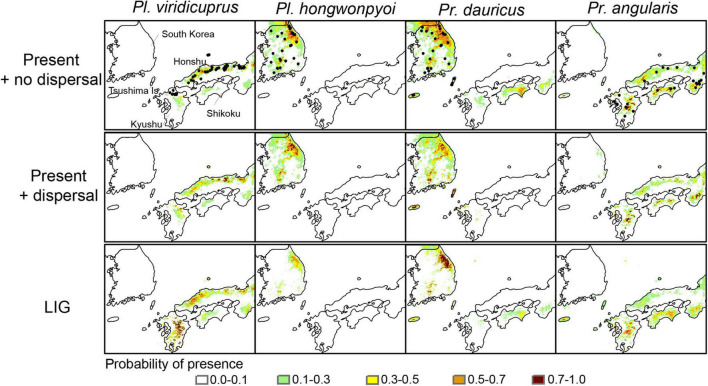
Locations of occurrences (black circles) and potentially suitable habitat for *Platycerus viridicuprus*, *Pl. hongwonpyoi*, *Prismognathus dauricus*, and *Pr. angularis* under present climate conditions (no dispersal, dispersal), the Last Interglacial (LIG) period in the study area. The colored areas represent moderate-to-high probability of species presence (>0.1).

Considering the known distribution of the four beetle species, we selected southern Japan and Korea (126°E–136°E, 31°N–38°N) as the geographical extent of our study (Figure 2). The occurrence data for *Platycerus viridicuprus* (*n* = 53), *Pl. hongwonpyoi* (*n* = 31), *Prismognathus dauricus* (*n* = 31), and *Pr. angularis* (*n* = 27) were obtained from field surveys and previous studies (Fujita, 2010; Imura, 2010; Kubota et al., 2011; Zhu et al., 2019, 2020a; Zhang and Kubota, 2021a; Figure 2).

Predictor variables were obtained from WorldClim with a spatial resolution of about 2.5 arc-min (v. 1.4; Hijmans et al., 2005).^1^ We then calculated pairwise Pearson’s correlation coefficients (*r*) and retained one predictor when two or more were highly correlated (i.e., | *r*| <0.80) (Lobo et al., 2010). When there were alternative candidate predictors to choose either, we gave priority to the predictor used in Homburg et al. (2013); Bosso et al. (2018), and Zhang and Kubota (2021a,b), which includes important predictors for *Platycerus* and other beetle species. In addition, we avoided Bio14 and Bio15 for past prediction, as it is biased when projected to past climate scenarios (Varela et al., 2015). Thus, we selected the following seven predictors for modeling: mean diurnal range (Bio2), isoterhermality (Bio3), maximum temperature of warmest month (Bio5), mean temperature of wettest quarter (Bio8), mean temperature of coldest quarter (Bio11), precipitation of wettest month (Bio13), and precipitation of driest quarter (Bio17) (Table 1 and Supplementary Table 4). We generated rasters for these variables at 2.5 arc-min for the Present time and at 30 arc-sec for the Last Interglacial (LIG; ∼120–140 ka) period. Notably, the last land-bridge between Japan and the Korean Peninsula disappeared in the beginning of LIG, they have not since been connected, including the Last Glacial Maximum (LGM) (Ohshima, 1990; Guedes et al., 2016).

**TABLE 1:** List of environmental variables used in the study.

Code	Environmental variables	Unit
Bio2	Mean diurnal range	°C
Bio3	Isothermality	−
Bio5	Max temperature of warmest month	°C
Bio8	Mean temperature of wettest quarter	°C
Bio11	Mean temperature of coldest quarter	°C
Bio13	Precipitation of wettest month	mm
Bio17	Precipitation of driest quarter	mm

We used SDMs for the four study species using MaxEnt v. 3.4.1, a machine learning presence−background algorithm, which is particularly suitable when absence data are not available (Phillips et al., 2006). The 10,000 background points were selected using the default setting. In MaxEnt, we used 70% of the occurrence data for model training and the remaining 30% of occurrence data for model evaluation. Ten replications were then chosen to run the model. The logistic output format was used for the model results, which indicates the habitat suitability of target species, and the resulting ASCII file was depicted using ArcGIS v. 10.4.1. Model performance was evaluated using the threshold independent area under the curve (AUC). AUC values of 1 indicate a perfect model based on the presence data of the samples, while a value of 0.5 indicates no difference from random model predictions (Phillips et al., 2006).

We also applied the null model approach for testing if the model performed significantly better than random expectation in the “dismo” package (Raes and ter Steege, 2007; Hijmans et al., 2017). A total of 100 different data sets with the same number of random points as the species occurrences in this study region was used to calculate the AUC for each species. We compared the AUC values with the ones calculated for a null model using Mann-Whitney *U*-tests.

Given that dispersal ability plays a key role in determining the distributional areas of species, we then conducted SDMs at the present time by incorporating the mid-range of locations (MR) dispersal constraint using the method of Zhang and Kubota (2021a). Here we used Euclidean distance to parameterize the dispersal kernel from the source point to the occurrence points of each species. A source point was defined by the mid-range longitude and latitude of the locations of each species (MR scenario). Although a SDM incorporating this scenario is effective to predict the species range of lucanid species in the present and future (Zhang and Kubota, 2021a), this modeling cannot be used for predictions in the past due to the possibility of recent local extinction.

In addition, we compared the raw MaxEnt outputs to evaluate niche similarity in their predicted niche distribution using ENMTools v. 1.4.4 (Warren et al., 2010). We calculated values of range overlap (Warren et al., 2008), Schoener’s *D* (Schoener, 1968), and Warren’s *I* index (Warren et al., 2008) among members of *Pl. viridicuprus* vs. *Pl. hongwonpyoi*, *Pl. viridicuprus* vs. *Pr. dauricus*, and *Pr. dauricus* vs. *Pr. angularis*. These indices range between 0 and 1, and values close to 1 indicate high similarity between the two compared groups.

### Differentiation in Ecological Space

To detect differentiation of the four study species in environmental space, we performed principal component analysis (PCA) to calculate principal components using the seven bioclimatic variables (Table 1) available at the Worldclim website. Then we extracted environmental values at the occurrence localities using ArcGIS v. 10.4.1 for each species. The statistical analysis was conducted using the R “stats” package v. 3.6.3 (R Core Team, 2013) with prcomp function and the result was visualized by function ggbiplot of “ggbiplot” package (Vu, 2011) and “ggplot2” (Wickham, 2007).

## Results

### Genetic Analyses

#### Yeast Isolation From Adult Females

We obtained yeast symbiont colonies from mycangial homogenates prepared from all *Prismognathus* females collected in Japan. The colonies on PDA plates were uniform, white, circular and protuberant, with a smooth edge and matte surface. The yeast CFU values ranged from 1.2 × 10^2^ to 2.7 × 10^4^ for *Prismognathus dauricus* and *Pr. angularis*.

#### Taxonomic Position of Yeast Symbionts Inferred From Internal Transcribed Spacer Phylogeny

**FIGURE 3:**
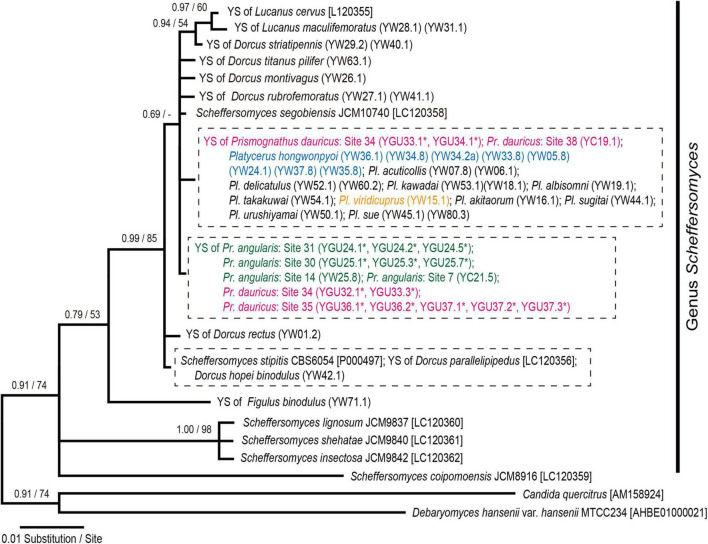
Bayesian inference (BI) phylogeny of the yeast symbionts of *Platycerus*, *Prismognathus*, and other stag beetles, and some yeast species based on ITS sequences. Numbers at the nodes indicate posterior probability for BI phylogeny (>50%)/bootstrap probability for maximum likelihood (ML) phylogeny (>50%). YS indicates yeast symbionts. Numbers following YW, YGU, or YC indicate the female code number and colony number (see Supplementary Table 2). Dashed boxes indicate ITS haplotypes shared by more than one species of the host stag beetles. Asterisks indicate the sequences determined in this study. HKY 85 + Gamma model (BI) and GTR + Gamma model (ML) were selected as the best-fit substitution models by Kakusan4. The BI and ML phylogenies are identical except for peripheral branches.

None of the ITS sequences of the yeast symbionts from the two *Prismognathus angularis* collected in Japan exhibited any sequence variation (containing partial 18S rRNA, ITS1, 5.8S rRNA, ITS2, and partial 26S rRNA; 651-bp; *n* = 6). These sequences were completely identical to sequences previously reported from two *Pr. angularis* specimens from Japan (Tanahashi et al., 2017; Zhu et al., 2020a). The ITS sequences for the yeast symbionts from three *Pr. dauricus* females (GU32, GU36, and GU37) collected in Tsushima also shared identical sequences (*n* = 7) with the yeast symbionts from *Pr. angularis*. By contrast, two sequences (652-bp) from a *Pr. dauricus* female (GU34) in Tsushima contained one nucleotide substitution and one insertion compared to those of the yeast symbionts from *Pr. angularis*. The latter sequences were identical to one Korean individual of *Pr. dauricus* (Zhu et al., 2020a) and the Korean and Japanese *Platycerus* species (Tanahashi et al., 2017; Kubota et al., 2020). Furthermore, the ITS sequences of eight yeast colonies isolated from one individual *Pr. dauricus* collected in Tsushima included both haplotypes: three yeast colonies were identical to the former, and the remaining five were identical to the latter. These sequences of yeast symbionts from *Prismognathus* and *Platycerus* species were similar to that of *Scheffersomyces segobiensis* (Santa-María and García-Aser, 1977), an already described yeast species that was first isolated from a jewel beetle *Chalcophora mariana massiliensis* (Coleoptera: Buprestidae) (Santa-María and García-Aser, 1977). In addition, the phylogenetic analysis based on the ITS sequences revealed that the yeast symbionts of the *Prismognathus* and *Platycerus* species constitute a distinct lineage to the clade of yeast symbionts of all other lucanid species, including environmental *Scheffersomyces* (Figure 3 and Supplementary Table 2). In total, 42 nucleotide substitutions and 12 insertions in the ITS were recognized among the yeast symbionts of Lucanid species and *Scheffersomyces* species.

#### Phylogeny of Yeast Symbionts Based on Inter-Genetic Spacer

The IGS sequences covering partial 26S rRNA, IGS1, 5S rRNA, IGS2, and partial 18S rRNA (2,184–2,195-bp; *n* = 16) were determined for *Prismognathus angularis* and *Pr. dauricus*. In total, 75 nucleotide substitutions and 23 insertions in the IGS were recognized. In *Pr. angularis*, the IGS sequences of eight colonies isolated from a host female exhibited 1–5 nucleotide substitutions and one insertion for the two females examined (GU24 and GU25), suggesting that genetic variation exists among the yeast symbionts in the mycangium of a single female. In *Pr. dauricus*, the IGS sequences of the eight colonies isolated from one host female were identical for the two females (GU32 and GU34) and exhibited two nucleotide substitutions and two insertions for the two females (GU36 and GU37) examined. Exceptionally, two different IGS sequences each contained 67 nucleotide substitutions and 10 insertions among the eight yeast colonies from one female (GU33) of *Pr. dauricus*.

**FIGURE 4:**
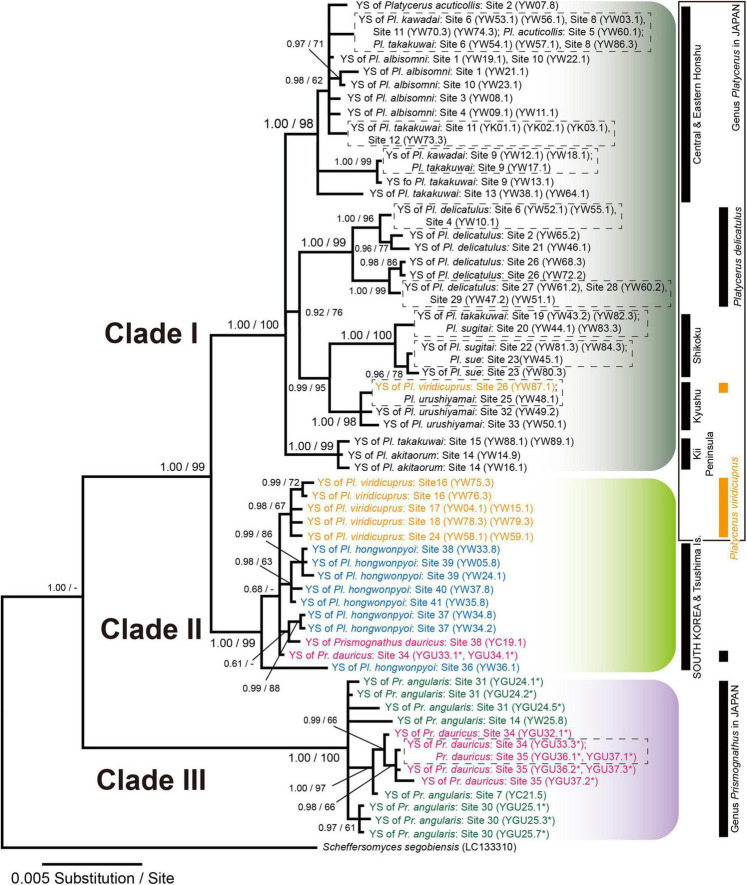
Bayesian inference (BI) phylogeny of the yeast symbionts of *Platycerus* and *Prismognathus* stag beetles based on IGS sequences. *Scheffersomyces segobiensis* was treated as the outgroup. Numbers at the nodes indicate posterior probability for BI phylogeny (>50%)/bootstrap probability for maximum likelihood (ML) phylogeny (>50%). YS indicates yeast symbionts. Numbers following YGU, YW, or YC indicate the female code number and colony number (see Supplementary Table 2). Dashed boxes indicate IGS haplotypes shared by more than one species of the host stag beetles. Asterisks indicate the sequences determined in this study. HKY 85 + Gamma model (BI) and GTR + Gamma model (ML) were selected as the best-fit substitution models by Kakusan4. The BI and ML phylogenies are identical except for peripheral branches.

In the IGS phylogeny, all yeast symbionts from all *Platycerus* species, one *Pr. dauricus* female in Tsushima and one *Pr. dauricus* female in South Korea, as well as a portion of the yeast symbionts from one *Pr. dauricus* female in Tsushima, formed a monophyletic clade (Figure 4). In this clade, two major subclades were recognized: Clade I contained primarily the yeast symbionts of the Japanese *Platycerus* species, whereas Clade II contained primarily those of the *Pl. viridicuprus* populations in Japan, all populations of *Pl. hongwonpyoi* in South Korea, two *Pr. dauricus* females in Tsushima and one *Pr. dauricus* female in South Korea. The yeast sequences from all *Pr. angularis* females collected in Japan and the four *Pr. dauricus* females in Tsushima formed another monophyletic clade (Clade III).

The yeast symbionts of Clades II (GU33, 34) and III (GU32, 33, 36, 37) were isolated from *Pr. dauricus* females in Tsushima. GU33 was the only female that possessed symbionts belonging to both Clades II and III (Figure 4 and Supplementary Table 2).

#### Phylogeny of Host Insects Based on Cytochrome Oxidase Subunit I and Comparison With Symbiont Phylogeny

**FIGURE 5:**
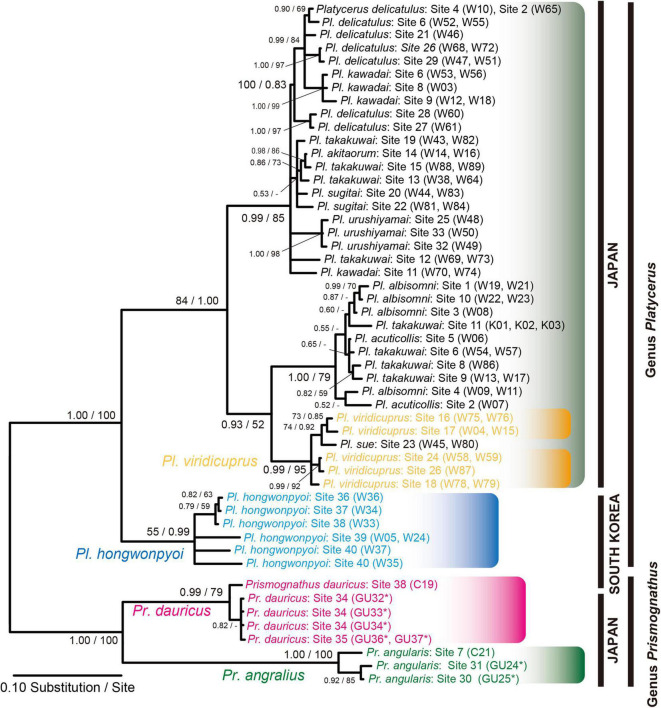
Bayesian inference (BI) phylogeny of *Platycerus* and *Prismognathus* stag beetles based on *COI* gene sequences. Numbers at the nodes indicate posterior probability for BI phylogeny (>50%)/bootstrap probability for maximum likelihood (ML) phylogeny (>50%). Asterisks indicate the sequences determined in this study. GU, W, or C indicate the female code number (see Supplementary Table 2). Asterisks indicate the sequences determined in this study. HKY 85 + Gamma Invariant model (BI) and GTR + Gamma model (ML) were selected as the best-fit substitution models by Kakusan4. The BI and ML phylogenies are identical except for peripheral branches.

We constructed the *COI* phylogeny of the host insects (Figure 5) and compared it to the IGS phylogeny of the yeast symbionts. Essentially, the host phylogeny and the symbiont phylogeny were not congruent, indicating that no strict host-symbiont co-speciation has been established in the evolutionary course of the *Prismognathus* and *Platycerus* stag beetles. The genera *Prismognathus* and *Platycerus* constituted an independent distant monophyletic group. Furthermore, *Pr. dauricus* constituted a different clade from that of *Pr. angularis*. Although 10 Japanese *Platycerus* species are distinctly identified by morphological features including genital organs (Kubota et al., 2009), introgressive hybridizations have occurred between some species combinations (e.g., *Pl. takakuwai*) (Figure 5). Such species always possess the Clade I yeasts in the IGS. *Pl. viridicuprus* forms a distinct monophyletic clade (Figure 5).

### Species Distribution Modeling

The mean AUC values for all the models and target species were 0.902–0.977; these models were significantly better than the null models, indicating that the models performed well (*p* < 0.001; Mann–Whitney *U*-test; Supplementary Table 5). The potential distributions of the four study species were well aligned with the known species’ ranges under present models with their respective dispersal constraints, although the potential area based on the models with no dispersal was larger than the areas for models incorporating dispersal constraints (Figure 2). For *Pl. hongwonpyoi* and *Pr. angularis*, the potential ranges during the LIG were smaller than those based on present climate conditions. By contrast, the MaxEnt models indicated a large potential range for *Pl. viridicuprus* in the LIG. In that time, the occurrences of *Pl. viridicuprus* in the Korean Peninsula and *Pl. hongwonpyoi* in Japan were not supported, whereas those of both *Pr. dauricus* and *Pr. angularis* in Kyushu, Japan, were predicted (Figure 2).

Range overlap and niche overlap analysis under present climatic conditions with no dispersal in the study region exhibited a pattern of little overlap for the SDMs of *Pl. hongwonpyoi* vs. *Pl. viridicuprus* and for *Pr. viridicuprus* vs. *Pr. dauricus*, but larger overlap for *Pr. dauricus* vs. *Pr. angularis* (Schoener’s *D* = 0.261, Warren’s *I* = 0.540, Range overlap = 0.318) (Table 2). In addition, the models that incorporated dispersal ability predicted the smallest overlap for all examined species pairs. During the LIG period, the lowest amount of overlap occurred between *Pl. hongwonpyoi* and *Pl. viridicuprus* (Schoener’s *D* = 0.029, Warren’s *I* = 0.111, Range overlap = 0), while the highest amount of overlap was between *Pr. dauricus* and *Pr. angularis* (Schoener’s *D* = 0.208, Warren’s *I* = 0.438, Range overlap = 0.306) (Table 2).

**TABLE 2:** Niche overlap (Schoener’s D, Warren’s I) values and range overlap for species-pair comparisons at Present and the Last interglacial (LIG) between *Platycerus* and *Prismognathus* species.

Species	Schoener’s *D*			Warren’s *I*			Range overlap		
	Present	Present	LIG	Present	Present	LIG	Present	Present	LIG
	+ no dispersal	+ dispersal		+ no dispersal	+ dispersal		+ no dispersal	+ dispersal	
*Pl. hongwonpyoi*	0.056	0.006	0.029	0.203	0.052	0.111	0.002	0	0
vs. *Pl. viridicuprus*									
*Pr. dauricus*	0.134	0.009	0.127	0.334	0.069	0.285	0.224	0	0.167
vs. *Pl. viridicuprus*									
*Pr. dauricus*	0.261	0.092	0.208	0.540	0.268	0.438	0.318	0.109	0.306
vs. *Pr. angularis*									

### Differentiation in Ecological Space

**FIGURE 6:**
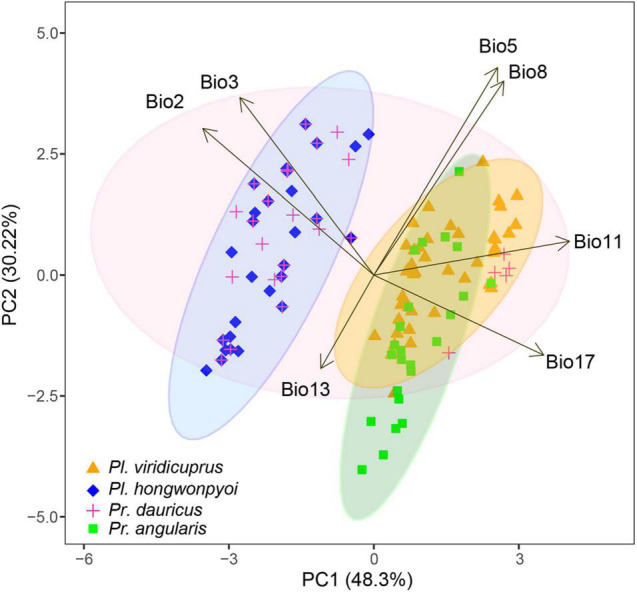
Principal component analysis (PCA) plots of the study species based on seven bioclimatic variables. Different colors indicate different species. Ellipses define 95% confidence intervals.

The potential distribution of the four species and their associated environmental variables can be estimated using the environmental PCA space (Figure 6 and Supplementary Table 6). The first two axes explained more than 78% of the variation in distribution. The climatic variables that contributed most to the first two principal components were mean temperature of coldest quarter (Bio11, 0.506 to PC1) and maximum temperature of warmest month (Bio5, 0.536 to PC2). In the environmental space, we found differentiation between *Pl. viridicuprus*, *Pl. hongwonpyoi*, and *Pr. dauricus*. However, the environment for *Pl. viridicuprus* was similar to that of *Pr. dauricus*, but more restricted. In addition, *Pr. dauricus* exhibited the largest environmental range.

## Discussion

The *Platycerus* species and their yeast symbionts co-evolved to some extent but incompletely based on their genetic phylogenies (Kubota et al., 2020). These results strongly suggest that lateral transfers and/or replacements of *Scheffersomyces* yeast symbionts are essentially rare but have occurred repeatedly during the evolutionary history of *Platycerus* (and probably also in other lucanid taxa).

To date, IGS sequence analyses have indicated that the yeast symbionts of Japanese and Korean *Platycerus* and *Prismognathus* possess the same monophyletic yeast symbionts [Clades (I + II + III), Figure 4]. This clade of symbionts is distinct from any other known yeast symbionts of other lucanid genera in East Asia (*Aegus*, *Dorcus*, *Prosopocoilus*, *Lucanus*, *Neolucanus*, *Figulus*, *Nigidius*, *Aesalus*, *Nicagus*, and *Ceruchus*: Tanahashi et al., 2010; Kubota et al., 2020; Zhu et al., 2020a; Figure 3), as stag beetles generally possess species- or genus-specific lineages of yeasts. That is, the lateral transmission of yeast symbionts between lucanid taxa is rare. The ancestor of all Japanese *Platycerus* species diverged from the ancestor of Asian continental *Platycerus* species, including *Pl. hongwonpyoi*, about 10 Mya (Zhu et al., 2020b). Compared to it, *Platycerus* and *Prismognathus* diverged earlier than 10 Mya (Figure 5). On the other hand, the yeast symbionts of Japanese *Pl. viridicuprus* covering a large area of species range (Sites 16, 17, 18, and 24: Supplementary Table 1) form a monophyletic clade, which is in a derived position compared to those of the Korean species in Clade II (Figures 4, 5). All other yeast symbionts are those of the Japanese *Platycerus* species belonging to Clade I (Figure 4). Therefore, to explain the phylogenetic discordance between the lucanid species and their yeast symbionts by the incomplete lineage sorting following ancestral polymorphism, the yeast IGS sequences within Clade II are too close (Figure 4). These findings suggest that the yeast symbionts were laterally transmitted from the Korean *Platycerus* or *Prismognathus* to *Pl. viridicuprus.*

This study presents novel results that a lucanid population (and a female) possesses two lineages of yeasts of different origins (Figures 3, 4). Based on IGS sequences at both the population and individual level (GU33), *Pr. dauricus* of the Tsushima Islands possesses yeast symbionts belonging to Clades II and III. Of these, the yeasts belonging to Clade III were closely related to and derived from those of Japanese *Pr. angularis* (Figure 4) and these two lucanid species seem to diverged earlier than 10 Mya (Figure 5), suggesting that the yeast symbionts (Clade III) were laterally transmitted from Japanese *Pr. angularis* to *Pr. dauricus* around the Tsushima Islands.

*Scheffersomyces* yeast symbionts can survive outside the symbiotic organ of the insect hosts and are cultivable (Supplementary Figure 2). These yeasts are commonly found in and have been isolated from the galleries of decaying wood inhabited by lucanid larvae based on our observations. The *Scheffersomyces* yeasts are involved in xylose fermentation and are thus likely to be adapted to the use of woody materials (Jeffries et al., 2007; Tanahashi et al., 2010). Meanwhile, the genetic lineages of the yeast symbionts corresponded to their host lucanid taxa (i.e., species or genus) (Figures 3, 4). These local host-symbiont compatibilities are likely due to the mycangium-mediated vertical transmission of the yeast symbionts generally found in stag beetles (Tanahashi et al., 2010). We have observed that *Platycerus* and *Prismognathus* species prefer similar mature temperate deciduous broad-leaved forests and similar white-rot wood. In fact, the adults or larvae of the two genera are often found in the same pieces of decaying wood in Japan and South Korea (Zhu et al., 2020a), which may lead to the lateral transmission of yeast symbionts among these taxa. It is unlikely that the yeast symbionts of Clades (I + II + III) were transmitted from other sympatric lucanid taxa to *Platycerus* or *Prismognathus* species, since the other taxa exclusively possess their own yeast lineages not but Clades (I + II + III) (Tanahashi et al., 2010; Kubota et al., 2020). Insects other than lucanid species may temporally possess and bring the yeast symbionts of Lucanid species. Even if yeast transmission between lucanid individuals via such other insects occurs, it is considered the same as transmission from a lucanid to a lucanid.

For yeast transmission between Japanese and Korean species to have occurred, they were likely sympatric in the past. However, *Pl. viridicuprus* and *Pr. angularis* in Japan are currently allopatrically distributed relative to *Pl. hongwonpyo*i (Korea) and *Pr. dauricus* (Korea and Tsushima in Japan, Figure 1). Therefore, estimations of the distributions of these species in the past are critical to the discussion of yeast transmission. Since *Platycerus* and *Prismognathus* prefer mature broad-leaved forests and showed fine geographical genetic variations due to their low dispersal ability (Figure 5; Kubota et al., 2011; Zhu et al., 2020a), they are unlikely to disperse across the sea. Because the last connection between Korea and Japan via the Tsushima land-bridge disappeared approximately 130 ka (the end of the Riss ice age) (Ohshima, 1990; Guedes et al., 2016), SDM projections for the target species in the LIG (∼120–140 ka) estimate the last sympatric possibilities.

Based on SDM projections, there is no potential range overlap between *Pl. viridicuprus* and *Pl. hongwonpyoi* both at present and in the LIG. Moreover, the environmental spaces did not overlap between these two species (Figure 2). These estimations suggest minimal possibility of lateral yeast transmission from *Pl. hongwonpyoi* to *Pl. viridicuprus*. Conversely, the SDM predicted larger range and niche overlaps between *Pr. dauricus* and *Pl. viridicuprus*, especially in the LIG (Figure 2 and Table 2). Their environmental spaces also largely overlapped (Figure 6). In the LIG, the range of *Pr. dauricus* was expected to extend to Japan, whereas that of *Pl. viridicuprus* was not expected to extend to the Tsushima Islands or South Korea (Figure 2). These findings suggest the possibility of lateral yeast transmission from *Pr. dauricus* to *Pl. viridicuprus* in Japan. The larger overlap of ecological space between these two species based on the PCA is consistent with this estimation (Figure 6). The ecological space of *Pr. dauricus* are separated to that in the Tsushima Islands and South Korea. Since the genetic differentiation of *P. dauricus* between both areas is less than those of *P. angularis*, *Pl. hongwonpyoi*, and *Pl. viridicuprus* within species (Figure 5), it is likely that *Pr. dauricus* has larger niche range than other species.

**FIGURE 7:**
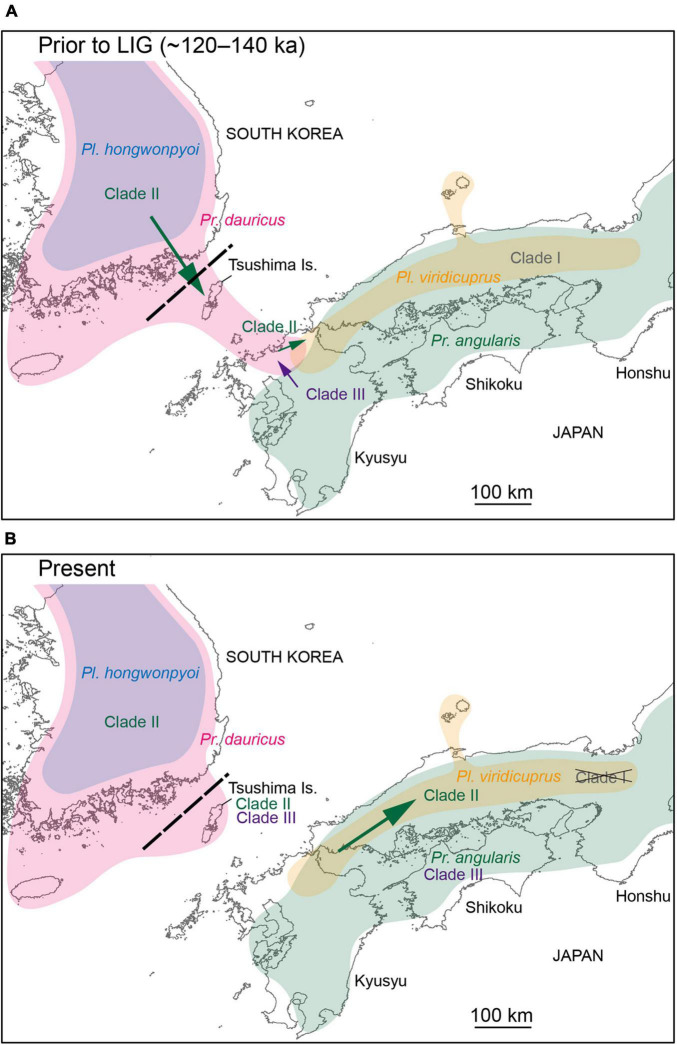
Schematic diagram of the lateral transmission of yeast symbionts among *Platycerus* and *Prismognathus* species in Japan and South Korea. During a certain period prior to the last interglacial (LIG) period, *Platycerus hongwonpyoi* and *Prismognathus dauricus* shared Clade II yeasts in South Korea. *Platycerus viridicuprus* and *Prismognathus angularis* possessed Clade I and Clade III yeasts, respectively, and did not share yeast symbionts in Japan. **(A)** Prior to or during the LIG, *Pr. dauricus* dispersed from Korea to Kyushu through the Tsushima Islands. Clade II yeasts were laterally transmitted from *Pr. dauricus* to *Pl. viridicuprus* in Kyushu. Meanwhile, Clade III yeasts were transmitted from *Pr. angularis* to *Pr. dauricus* in Kyushu (or on the Tsushima Islands, if *Pr. angularis* had reached there at that time). **(B)** At the present time, Clade I yeasts have been nearly replaced by Clade II yeasts in *Pl. viridicuprus* in Kyushu and western Honshu. On the Tsushima Islands, Clade II yeasts are being replaced by Clade III yeasts, or they coexist in *Pr. dauricus*.

Additionally, the yeast symbionts of Clade III are likely to have been transmitted from *Pr. angularis* to *Pr. dauricus* (Figure 4). For these two species, the potential range and niche overlaps were relatively high all in the present, LIG (Figure 2 and Table 2). Because *Pr. angularis* and *Pr. dauricus* are actually allopatric, range exclusion appears to exist between them, perhaps caused by interspecific competition or reproductive interference. If so, it is possible that these two species have been in contact with each other in Tsushima or Kyushu, which is close to the current boundary. Since *Pl. viridicuprus* hardly reached Tsushima in the LIG, *Pr. dauricus* likely existed in Kyushu then. Our hypotheses are summarized in Figure 7: the Clade II yeasts were likely transmitted from *Pr. dauricus* to *Pl. viridicuprus* in Kyushu, and the Clade III yeasts were transmitted from *Pr. angularis* to *Pr. dauricus* around Tsushima. For the yeast symbionts of *Pr. dauricus* in Tsushima, replacement from Clade II to Clade III may be occurring.

Although 10 *Platycerus* species, including *Pl. viridicuprus* and *Pr. angularis* are widely sympatric in Japan, there is no *Platycerus* species possessing Clade III yeasts (*n* = 67) or *Pr. angularis* possessing Clade I or II yeasts (*n* = 7) (Tanahashi et al., 2010, 2017; Kubota et al., 2020; Zhu et al., 2020a). Therefore, yeast transmission between *Platycerus* and *Pr. angularis* is unlikely to occur. In addition, *Pl. viridicuprus* is distributed sympatrically with *Pl. delicatulus* and parapatrically with *Pl. urushiyamai* and *Pl. takakuwai* in western Japan, whereas Japanese *Platycerus* other than *Pl. viridicuprus* always possess the Clade I yeasts and not Clade II yeasts, even if they are collected from the same wood piece. By contrast, some Japanese *Platycerus* species share the same lineage of yeasts. These facts indicate that the presence or absence of the yeast transmission is varied even among closely related species. However, the benefit of yeast replacement for the host lucanid species and yeasts is still unclear.

Applications of SDMs to paleobiology have grown substantially during the last decade due to their accessibility and the improved availability of paleoclimate simulations (Svenning et al., 2011; Gavin et al., 2015). Specifically, previous research using SDM typically had several main objectives: to understand past shifts of species with climate; to identify glacial refugia, particularly during the cold period of the Last Glacial Maximum (LGM, ∼21 ka); and to determine the effect of climate and interspecific interactions in moving hybrid zones (Engler et al., 2013; Rödder et al., 2013; Sosa-Pivatto et al., 2017; Coelho et al., 2019). However, the LIG projections in the present study have provided insight into microbial transmission among the study species. We also present novel estimates of the lateral transmission process of microbial symbionts based on phylogenetic, SDM and environmental analyses among lucanid beetles. Such elucidation of the yeast transmission as in this study will contribute to our understanding of the evolutionary dynamics of insect–microbial symbiotic systems, especially the impacts to co-evolutionary relationships.

## Data Availability Statement

The sequence datasets presented in this study can be found in the online repository of DNA Data Bank of Japan (DDBJ). The accession numbers can be found in Supplementary Table 2.

## Author Contributions

GU, S-NZ, and X-JZ were interested in evolutionary ecology of stag beetles and their yeast symbionts. GU contributed to the data generation of this study and performed the genetic analyses. S-NZ performed the SDM and ecological space analysis. KK contributed to the study design. GU and S-NZ wrote the manuscript under the supervising of KT and KK. All authors approved the final version of the manuscript.

## Conflict of Interest

The authors declare that the research was conducted in the absence of any commercial or financial relationships that could be construed as a potential conflict of interest.

## Publisher’s Note

All claims expressed in this article are solely those of the authors and do not necessarily represent those of their affiliated organizations, or those of the publisher, the editors and the reviewers. Any product that may be evaluated in this article, or claim that may be made by its manufacturer, is not guaranteed or endorsed by the publisher.

## Footnotes

1
http://www.worldclim.org
