# Supplementary material for: Lateral Transmission of Yeast Symbionts Among Lucanid Beetle Taxa
Source: Front Microbiol. 2021 Dec 14;12:794904. doi: 10.3389/fmicb.2021.794904 (PMC8712881; doi:10.3389/fmicb.2021.794904)
Supplement: Supplementary file 1 [file Data_Sheet_1.PDF]

Supplementary Table 1. Sample collection Sites.

Site	Locality	Altitude	Latitude	Longitude
	Japan			
1	Yumiharidaira, Nishikawa-machi, Yamagata Pref.	640	38.48	140.01
2	Mt. Takayama, Nikko-shi, Tochigi Pref.	1280	36.75	139.44
3	Mt.Akagunayama, Fujioka-shi, Gunma Pref.	1100	36.15	138.90
4	Irikawa, Chichibu-shi, Saitama Pref.	1300	35.94	138.80
5	Kubo, Dôshi-mura, Yamanashi Pref.	460	35.54	139.09
6	Mt. Kanyudoyama, Yamakita-chô, Kanagawa Pref.	1400	35.51	139.05
7	Mt. Mikuniyama, Kanagawa Pref.	1280	35.40	138.92
8	Mt. kamiyama, Hakone-chô, Kanagawa Pref.	1420	35.23	139.02
9	Haccho-ike, Izu-shi, Shizuoka Pref.	1200	34.85	138.96
10	Mt. Kenashiyama, Nozawaonsen-mura, Nagano Pref.	1330	36.91	138.48
11	Mt. Torikurayama, Ôshika-mura, Nagano Pref.	1640	35.55	138.09
12	Mt. Funayama, Takayama-shi, Gifu Pref.	1470	36.02	137.24
13	Mt. Ôborayama, Tsu-shi, Mie Pref.	770	34.53	136.22
14	Mt. Nagoyadake, Ôdaigaharayama, Ôdai-chô, Mie Pref.	1520	34.19	136.10
15	Mt. Obagamine, Kamikitayama-mura, Nara Pref.	1100	34.23	136.02
16	Mt. Ôginosen, Shin-onsen-chô, Hyôgo Pref.	1000	35.44	134.46
17	Mt. Washigamine, Okinoshima-chô, Shimane Pref.	450	36.26	133.33
18	Mt. Tachieboshiyama, Shôbara-shi, Hiroshima Pref.	1180	35.05	133.07
19	Dosu-tôge Pass, Kamiyama-chô, Tokushima Pref.	1030	33.92	134.29
20	Mt. Marusasayama, Mima-shi, Tokushima Pref.	1370	33.87	134.09
21	Mt. Tsurugisan, Miyoshi-shi, Tokushima Pref.	1320	33.87	134.09
22	Mt. Ishizuchisan, Saijo-shi, Ehime Pref.	1430	33.75	133.15
23	Mt. Ishizuchisan, Saijo-shi, Ehime Pref.	1680	33.77	133.12
24	Mt. Fukuchiyama, Nôgata-shi, Fukuoka Pref.	680	33.75	130.80
25	Mt. Hikosan, Hikosan, Soeda-machi, Fukuoka Pref.	960	33.48	130.93
26	Mt. Gakumekisan, Soeda-machi, Fukuoka Pref.	740	33.46	130.91
27	Mt. Yufudake, Beppu-shi, Ôita Pref.	1100	33.28	131.40
28	Mt. Kurodake, Yufu-shi, Ôita Pref.	880	33.12	131.29
29	Mt. Gokaharadake, Ômura-shi, Nagasaki Pref.	900	32.96	130.08
30	Mt. Unzendake, Unzen-shi, Nagasaki Pref.	1100	32.76	130.28
31	Mt. Ôyanodake, Asogun Minamiasomura, Kumamoto	1100	32.79	131.01
32	Mt. Shiratoriyama, Yatsushiro-shi, Kumamoto Pref.	1480	32.48	131.00
33	Mt. Shiragadake, Asagiri-machi, Kumamoto Pref.	1370	32.16	130.94
34	Mt. Taterasan, Tsushima-shi, Nagasaki Pref.	230	34.15	129.22
35	Mt. Mitake, Tsushima-shi, Nagasaki Pref.	370	34.58	129.38
	South Korea			

36	Mt. Hangeryeoung, Gangwon Province I	950	38.09	128.41
37	Mt. Jengoge Pass, Gangwon Prov. II	650	37.79	128.62
38	Birosa, Gyensanbuk Prov.	600	36.94	128.50
39	Mt. Deogyusan, Jeollabuk Prov. I	1600	35.86	127.75
40	Mt. Jeoksangsan, Jeollabuk Prov. II	500	35.97	127.70
41	Mt. Nogodan, Jeollanam Prov.	1430	35.30	127.53

Sites 30-31 and 34-35 are collection sites in this study. Sites 1–6, 8-29, 32-33 from Kubota et al. (2020); Sites 7 and 38 from from Zhu et al. (2020); Sites 36-41 from Tanahashi et al. (2017).
