# Supplementary material for: Lateral Transmission of Yeast Symbionts Among Lucanid Beetle Taxa
Source: Front Microbiol. 2021 Dec 14;12:794904. doi: 10.3389/fmicb.2021.794904 (PMC8712881; doi:10.3389/fmicb.2021.794904)
Supplement: Supplementary file 2 [file Data_Sheet_2.PDF]

Supplementary Table 2. Yeast and insect samples examined.

Host insect species	Site	Host female no.	Yeast: Strain (number of clones examined) ITS	Yeast: Strain (number of clones examined) IGS	Yeast: GenBank accession no. ITS	Yeast: GenBank accession no. IGS	Insect sequence: GenBank accession no. *COI*
Prismognathus dauricus Motschulsky, 1860	34	GU32	YGU32.1(8)	YGU32.1(8)	LC631316	LC631331	LC631346
	34	GU33	YGU33.1(5)	YGU33.1(5)	LC631317	LC631332	LC631347
			YGU33.3(3)	YGU33.3(3)	LC631318	LC631333	
	34	GU34	YGU34.1(8)	YGU34.1(8)	LC631319	LC631334	LC631348
	35	GU36	YGU36.1(6)	YGU36.1(6)	LC631320	LC631335	LC631349
			YGU36.2(2)	YGU36.2(2)	LC631321	LC631336	
	35	GU37	YGU37.1(6)	YGU37.1(6)	LC631322	LC631337	LC631350
			YGU37.2(1)	YGU37.2(1)	LC631323	LC631338	
			YGU37.3(1)	YGU37.3(1)	LC631324	LC631339	
	38	C19	YC19.1(1)	YC19.1(1)	(LC492883)****	(LC492885)****	(LC492881)****
Prismognathus anguralis Waterhouse, 1874	31	GU24	YGU24.1(1)	YGU24.1(1)	LC631325	LC631340	LC631351
			YGU24.2(5)	YGU24.2(5)	LC631326	LC631341	
			YGU24.5(2)	YGU24.5(2)	LC631327	LC631342	
	30	GU25	YGU25.1(4)	YGU25.1(4)	LC631328	LC631343	LC631352
			YGU25.3(3)	YGU25.3(3)	LC631329	LC631344	
			YGU25.7(1)	YGU25.7(1)	LC631330	LC631345	
	7	C21	YC21.5(1)	YC21.5(1)	(LC492884)****	(LC492886)****	(LC492882)****
	14	W25	YW25.8(3)	YW25.8(8)	(LC133298)*	(LC133309)*	
Platycerus delicatulus Lewis, 1883	2	W65		YW65.2(2)		(LC438669)***	(LC438728)***
	4	W10		YW10.1(2)		(LC438670)***	(AB426945)**
	6	W52	YW52.1(1)	YW52.1(2)	(LC133297)*	(LC133308)*	(LC133287)*
	6	W55		YW55.1(2)		(LC438671)***	
	21	W46		YW46.1(2)		(LC438672)***	(AB426954)**
	26	W68		YW68.3(4)		(LC438673)***	(LC438729)***
	26	W72		YW72.2(4)		(LC438674)***	
	27	W61		YW61.2(2)		(LC438675)***	(AB609396)**
	28	W60	YW60.2(2)	YW60.2(2)	(LC438646)***	(LC438676)***	(AB426955)**
ssp. *unzendakensis* Fujita et Ichikawa, 1982	29	W47		YW47.2(3)		(LC438677)***	(LC438730)***
	29	W51		YW51.1(2)		same as LC438677	
P. kawadai Fujita et Ichikawa, 1982	6	W53	YW53.1(2)	YW53.1(2)	(LC438647)***	(LC438678)***	(LC438731)***
	6	W56		YW56.1(2)		(LC438679)***	
	8	W03		YW03.1(3)		(LC438680)***	(LC438732)***
	9	W12		YW12.1(3)		(LC438681)***	(LC438733)***
	9	W18	YW18.1(2)	YW18.1(2)	(LC438648)***	(LC438682)***	
	11	W70		YW70.3(4)		(LC438683)***	(LC438734)***
	11	W74		YW74.3(4)		(LC438684)***	
P. acuticollis K. Kurosawa, 1969	2	W07	YW07.8(1)	YW07.8(6)	(LC133296)*	(LC133307)*	(LC133286)*
	5	W06	YW06.1(2)	YW06.1(4)	(LC438649)***	(LC438685)***	(LC438735)***
P. albisomni Kubota, Kubota et Otobe, 2008	1	W19	YW19.1(2)	YW19.1(3)	(LC438650)***	(LC438686)***	(AB426970)**
	1	W21		YW21.1(3)		(LC438687)***	
	10	W22		YW22.1(3)		(LC438688)***	(LC438736)***
	10	W23		YW23.1(2)		(LC438689)***	
ssp. *chichibuensis* Kubota, Kubota et Otobe, 2008	3	W08		YW08.1(2)		(LC438690)***	(AB573678)**
	4	W09		YW09.1(3)		(LC438691)***	(AB426987)**
	4	W11		YW11.1(3)		(LC438692)***	
P. takakuwai Fujita, 1987	6	W54	YW54.1(2)	YW54.1(2)	(LC438651)***	(LC438693)***	(LC438737)***
	6	W57		YW57.1(2)		(LC438694)***	

	8	W86		YW86.3(3)		(LC438695)***	(LC438738)***
	9	W13		YW13.1(3)		(LC438696)***	(LC438739)***
	9	W17		YW17.1(2)		(LC438697)***	
	11	K01		YK01.1(8)		(LC438698)***	
	11	K02		YK02.1(8)		(LC438699)***	(LC438740)***
	11	K03		YK03.1(8)		(LC438700)***	
ssp. *akitai* Fujita, 1987	12	W69	No colony	No colony			(AB609474)**
	12	W73		YW73.3(4)		(LC438701)***	
	13	W38		YW38.1(2)		(LC438702)***	(LC438741)***
	13	W64		YW64.1(2)		(LC438703)***	
	15	W88		YW88.1(8)		(LC438704)***	(LC438742)***
	15	W89		YW89.1(8)		(LC438705)***	
ssp. *namedai* Fujita, 1987	19	W43		YW43.2(2)		(LC438706)***	(AB427025)**
	19	W82		YW82.3(4)		(LC438707)***	
P. viridicuprus Kubota, Kubota et Otobe, 2008	16	W75		YW75.3(4)		(LC438708)***	(AB427017)**
	16	W76		YW76.3(4)		(LC438709)***	
	17	W04		YW04.1(3)		(LC438710)***	(LC438743)***
	17	W15	YW15.1(2)	YW15.1(2)	(LC438652)***	(LC438711)***	
	18	W78		YW78.3(4)		(LC438712)***	(AB609531)**
	18	W79		YW79.3(4)		(LC438713)***	
ssp. *kanadai* Kubota, Kubota et Otobe, 2008	24	W58		YW58.1(2)		(LC438714)***	(AB427032)**
	24	W59		YW59.1(2)		(LC438715)***	
	26	W87		YW87.1(8)		(LC438716)***	(LC438744)***
P. akitaorum Imura, 2007	14	W14		YW14.9(3)		(LC438717)***	(AB427035)**
	14	W16	YW16.1(2)	YW16.1(2)	(LC438653)***	(LC438718)***	
P. sugitai Okuda et Fujita, 1987	20	W44	YW44.1(2)	YW44.1(2)	(LC438654)***	(LC438719)***	(AB588794)**
	20	W83		YW83.3(4)		(LC438720)***	
	22	W81		YW81.3(4)		(LC438721)***	(AB588806)**
	22	W84		YW84.3(4)		(LC438722)***	
P. urushiyamai Imura, 2007	25	W48		YW48.1(2)		(LC438723)***	(AB427045)**
	32	W49		YW49.2(2)		(LC438724)***	(LC438745)***
	33	W50	YW50.1(1)	YW50.1(2)	(LC438655)***	(LC438725)***	(AB609567)**
P. sue Imura, 2007	23	W45	YW45.1(2)	YW45.1(2)	(LC438656)***	(LC438726)***	(AB588778)**
	23	W80	YW80.3(2)	YW80.3(4)	(LC438657)***	(LC438727)***	
P. hongwonpyoi Imura et Choe, 1989	36	W36	YW36.1(1)	YW36.1(7)	(LC133288)*	(LC133299)*	(LC133280)*
(from South Korea)	37	W34	YW34.8(1)	YW34.8(5)	(LC133289)*	(LC133300)*	(LC133281)*
			YW34.2a(1)	YW34.2a(3)	(LC133290)*	(LC133301)*	
	38	W33	YW33.8(3)	YW33.8(8)	(LC133291)*	(LC133302)*	(LC133282)*
	39	W05	YW05.8(1)	YW05.8(8)	(LC133292)*	(LC133303)*	(LC133283)*
		W24	YW24.1(1)	YW24.1(4)	(LC133293)*	(LC133304)*	
	40	W37	YW37.8(1)	YW37.8(8)	(LC133294)*	(LC133305)*	(LC133284)*
	41	W35	YW35.8(1)	YW35.8(8)	(LC133295)*	(LC133306)*	(LC133285)*
Lucanus cervus (Linnaeus, 1758)					(LC120355)		
L. maculifemoratus Motsulsky, 1861		W28	YW28.1(2)		(LC438658)***		
		W31	YW31.1(2)		(LC438659)***		
Dorcus parallelipipedus (Linnaeus, 1758)					(LC120356)		
D. striatipennis (Motchulsky, 1861)		W29	YW29.2(2)		(LC438660)***		
		W40	YW40.1(2)		(LC438661)***		
D. rubrofemoratus (Vollenhoven, 1865)		W27	YW27.1(2)		(LC438662)***		
		W41	YW41.1(2)		(LC438663)***		
D. rectus (Motchulsky, 1857)		W01	YW01.2(2)		(LC438664)***		

D. montivagus (Lewis, 1883)	W26	YW26.1(2)	(LC438665)***		
D. hopei binodulosus Waterhouse, 1874	W42	YW42.1(2)	(LC438666)***		
D. titanus pilifer Vollenhoven, 1861	W63	YW63.1(2)	(LC438667)***		
Figulus binodulus Waterhouse, 1873	W71	YW71.1(2)	(LC438668)***		
(Yeast Species)					(Source)
Sheffersomyces segobiensis (Santa Maria & C. Garcia)		JCM10740	(LC120358)	(LC133310)	*Calcophora Mariana*
S. stipitis (Pignal)		CBS6054	(CP000497)		?
S. coipomensis (Ramirez & Gonzalez)		JCM8916	(LC120359)		Fallen trunk of *Eucryphia cordifolia*
S. lignosum (Kurtzman)		JCM9837	(LC120360)		Rotten wood
S. shehatae (H.R. Buckley & van Uden)		JCM9840	(LC120361)		Dead insect invaded pine tree
S. insectosa (Kurtzman)		JCM9842	(LC120362)		*Leptura maculicornis*
Candida quercitrusa S. A. Meyer & Phaff		CBS4412	(AM158924)		Insect frass on an oak tree
Debaryomyces hansenii var. *hansenii* (Zopf) Lodder & Kreger-van Rij		MTCC_234	(AHBE01000021)		Soil

Accession numbers in parentheses have already been published in previous studies. *, from Tanahashi et al. (2017). **, from Kubota et al. (2010); Kubota & Kubota (2011); Kubota et al. (2011). ***, from Kubota et al. (2020). ****, from Zhu et al. (2020). JCM, Japan Collection of Microorganisms, RIKEN BioResource Research Center, Tsukuba, Japan; CBS, Centraal Bureau voor Schimmelcultures, Utrecht, the Netherland; MTCC, Microbial Type Culture Collection and Gene Bank, CSIR-Institute of Microbial Technology, Chandiarh, India.
