# Supplementary material for: Lateral Transmission of Yeast Symbionts Among Lucanid Beetle Taxa
Source: Front Microbiol. 2021 Dec 14;12:794904. doi: 10.3389/fmicb.2021.794904 (PMC8712881; doi:10.3389/fmicb.2021.794904)
Supplement: Supplementary file 3 [file Data_Sheet_3.PDF]

Supplementary Table 3. ITS, IGS, and *COI* primers used in this study.

Organism	Region	Name	Sequence (5'-3')	Strand direction	Usage*	Reference
Scheffersomyces	ITS	NS7	GAGGCAATAACAGGTCTGTGATGC	Forward	P, S	White et al. (1990)
		ITS5	GGAAGTAAAAGTCGTAACAAGG	Forward	S	White et al. (1990)
		NL4	GGTCCGTGTTTCAAGACGG	Reverse	P	White et al. (1990)
	IGS	IGS1	GCCTTGTTGTTACGATCTGC	Forward	P, S	Tanahashi et al. (2017)
		IGS2	ACCGTTTCCCGTCCGATCAAC	Reverse	S	Tanahashi et al. (2017)
		IGS3	TCCCACTACACTACTCGGTC	Forward	S	Tanahashi et al. (2017)
		IGS4	GAGACAAGCATATGACTAC	Reverse	P, S	Tanahashi et al. (2017)
		IGS7i	GAAGAGAGTTTAATGGTGAAC	Forward	S	Tanahashi et al. (2017)
		IGS8i	GTTCACCATTAAACTCTCTCC	Reverse	S	Tanahashi et al. (2017)
Lucanidae	*COI*	C1-J-2183	CAACATTTATTTTGATTTTTTGG	Forward	P, S	Simon et al. (1994)
		L2-N-3014	TCCAATGCACTAATCTGCCATATTA	Reverse	P, S	Simon et al. (1994)

*: P, PCR; S, sequencing analysis.
