# Supplementary material for: Lateral Transmission of Yeast Symbionts Among Lucanid Beetle Taxa
Source: Front Microbiol. 2021 Dec 14;12:794904. doi: 10.3389/fmicb.2021.794904 (PMC8712881; doi:10.3389/fmicb.2021.794904)
Supplement: Supplementary file 4 [file Data_Sheet_4.PDF]

Supplementary Table 4. Pairwise Person correlation coefficients for climatic variables used in species distribution modeling of studied species.

	Bio2	Bio3	Bio5	Bio8	Bio11	Bio13	Bio17
Bio2	1						
Bio3	0.44	1					
Bio5	-0.18	0.15	1				
Bio8	0.00	0.12	0.53	1			
Bio11	-0.76	0.11	0.64	0.29	1		
Bio13	0.15	-0.07	-0.31	-0.07	-0.26	1	
Bio17	-0.76	-0.07	0.21	-0.15	0.71	-0.13	1
