# Supplementary material for: Lateral Transmission of Yeast Symbionts Among Lucanid Beetle Taxa
Source: Front Microbiol. 2021 Dec 14;12:794904. doi: 10.3389/fmicb.2021.794904 (PMC8712881; doi:10.3389/fmicb.2021.794904)
Supplement: Supplementary file 5 [file Data_Sheet_5.PDF]

**Supplementary Table 5.** The mean value of area under the curve (AUC) for real model and null model for each species.

| Species | Sample size | Model | Real model AUC | Null model AUC |
|---|---|---|---|---|
| *Platycerus viridicuprus* | 53 | No dispersal | 0.977*** | 0.708 |
| | | Dispersal | 0.974*** | 0.662 |
| *Pl. hongwonpyoi* | 31 | No dispersal | 0.949*** | 0.734 |
| | | Dispersal | 0.953*** | 0.703 |
| *Prismognathus dauricus* | 31 | No dispersal | 0.902*** | 0.631 |
| | | Dispersal | 0.949*** | 0.700 |
| *Pr. angularis* | 27 | No dispersal | 0.949*** | 0.640 |
| | | Dispersal | 0.966*** | 0.686 |

***, performed significantly better than null model ($p<0.001$, Mann–Whitney U-test).
