# Supplementary material for: Lateral Transmission of Yeast Symbionts Among Lucanid Beetle Taxa
Source: Front Microbiol. 2021 Dec 14;12:794904. doi: 10.3389/fmicb.2021.794904 (PMC8712881; doi:10.3389/fmicb.2021.794904)
Supplement: Supplementary file 6 [file Data_Sheet_6.PDF]

Supplementary Table 6. Principal components analysis (PCA) loading scores from environmental predictors for the first two axes used to estimate variance among four species (*Platycerus viridicuprus* , *Pl. hongwonpyoi* , *Prismognathus dauricus* , *Pr. angularis*).

Variables	Code	PC1	PC2
Mean Diurnal Range	Bio2	-0.442	0.378
Isothermality	Bio3	-0.347	0.458
Max Temperature of Warmest Month	Bio5	0.32	**0.536**
Mean Temperature of Wettest Quarter	Bio8	0.336	0.501
Mean Temperature of Coldest Quarter	Bio11	**0.506**	0.088
Precipitation of Wettest Month	Bio13	-0.137	-0.242
Precipitation of Driest Quarter	Bio17	0.44	-0.207
	% variance	48.30	30.22

The variable that contributed the most is highlighted in bold on each axis.
