# Supplementary material for: Lateral Transmission of Yeast Symbionts Among Lucanid Beetle Taxa
Source: Front Microbiol. 2021 Dec 14;12:794904. doi: 10.3389/fmicb.2021.794904 (PMC8712881; doi:10.3389/fmicb.2021.794904)
Supplement: Supplementary file 7 [file Data_Sheet_7.PDF]

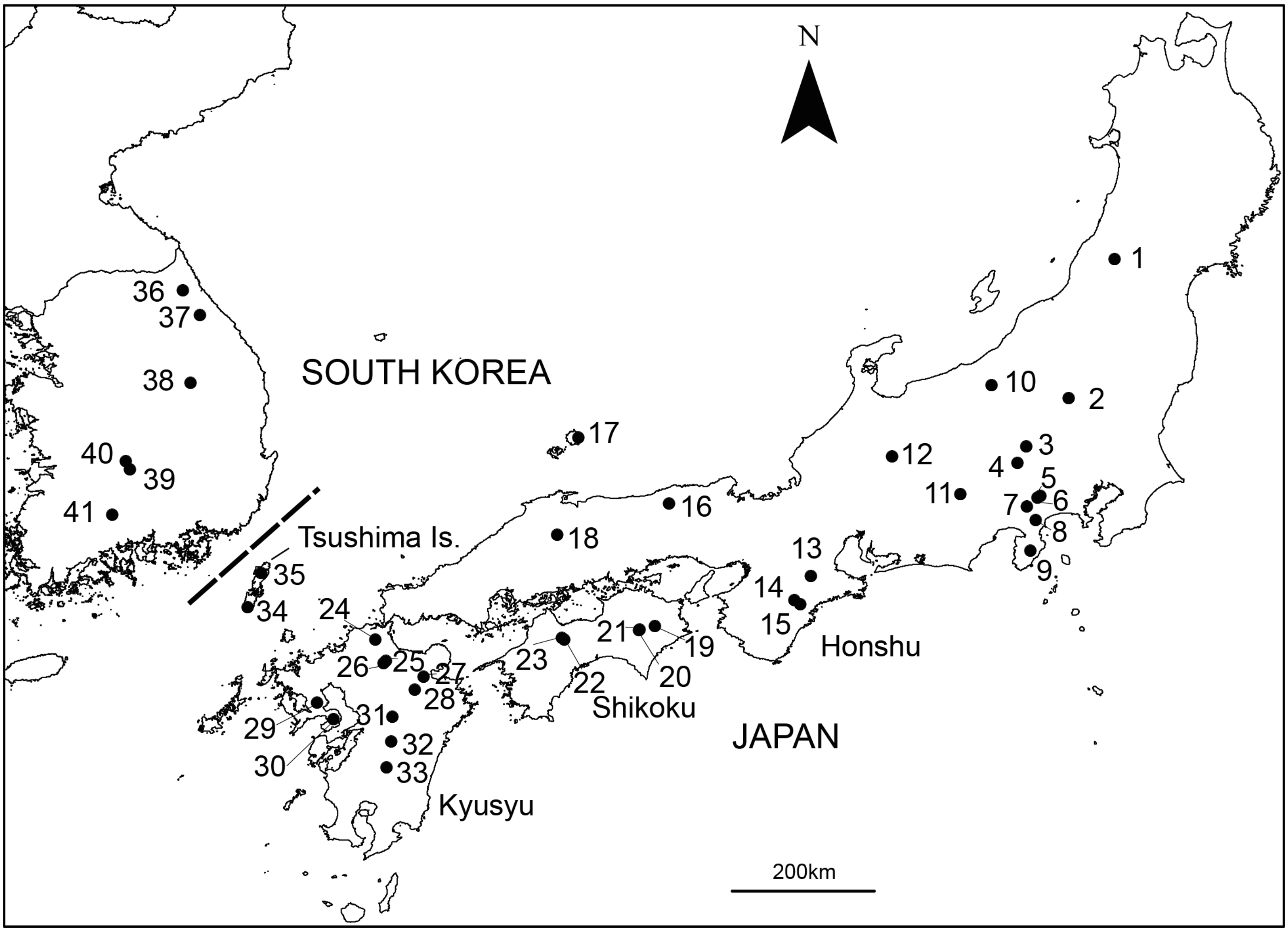

Supplementary Figure 1. Lucanid sample collection sites for the yeast extraction (including the sites in this study, Kubota et al., 2020 and Zhu et al., 2020).
