# Supplementary material for: Lateral Transmission of Yeast Symbionts Among Lucanid Beetle Taxa
Source: Front Microbiol. 2021 Dec 14;12:794904. doi: 10.3389/fmicb.2021.794904 (PMC8712881; doi:10.3389/fmicb.2021.794904)
Supplement: Supplementary file 8 [file Data_Sheet_8.PDF]

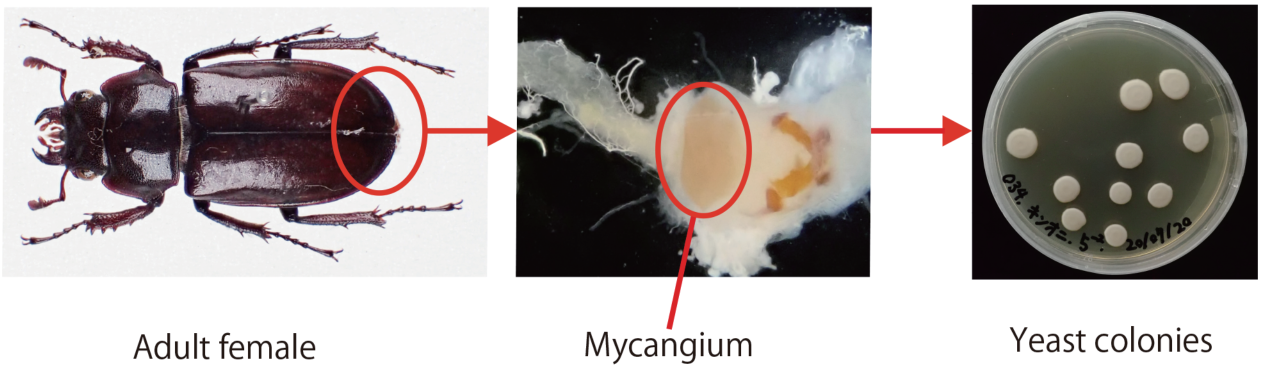

Supplementary Figure 2. Adult female, dissected mycangium and isolated yeast colonies of *Prismognathus dauricus*.
