# Supplementary material for: Lateral Transmission of Yeast Symbionts Among Lucanid Beetle Taxa
Source: Front Microbiol. 2021 Dec 14;12:794904. doi: 10.3389/fmicb.2021.794904 (PMC8712881; doi:10.3389/fmicb.2021.794904)
Supplement: Supplementary file 9 [file Data_Sheet_9.PDF]

Supplementary Appendix 1.

Protocol of yeast isolation from adult females (based on Kubota et al., 2020)

A mycangium was dissected from each adult female in sterilized phosphate-buffered saline (PBS) as described previously (Tanahashi et al., 2017). The dissected mycangium was homogenized in PBS using a pellet pestle in a plastic tube, and each of a 5-fold dilution series of the homogenate (equivalent to $1/5^1$, $1/5^2$, …, $1/5^5$ of the dissected mycangium) was spread onto a potato dextrose agar (PDA: BIOKAR Diagnostics, Rue des 40 Mines, 60000 Allonne, France) plate containing 20 μL/mL rifampicin. The plates were incubated at 20°C for 4 days. The number of colony-forming units (CFU) per organ was calculated based on plates for which an adequate number (usually 30~500) of colonies appeared (Supplementary Figure 2).
