# Supplementary material for: Lateral Transmission of Yeast Symbionts Among Lucanid Beetle Taxa
Source: Front Microbiol. 2021 Dec 14;12:794904. doi: 10.3389/fmicb.2021.794904 (PMC8712881; doi:10.3389/fmicb.2021.794904)
Supplement: Supplementary file 10 [file Data_Sheet_10.PDF]

Supplementary Appendix 2.

Protocol of determining DNA sequences of yeast symbionts

Because no morphological differences were observed among yeast colonies that appeared on individual plates, we randomly selected eight colonies for each host individual and transferred them to new PDA plates for subsequent DNA analyses. A small pellet of each yeast colony was suspended in 50 µL of lyticase solution (0.4 U/µL lyticase, 50 mM ethylenediaminetetraacetic acid [EDTA]) and incubated at 37°C for 2 h. The cell suspension was discarded. The yeast DNA was extracted and purified as described (Tanahashi et al. 2017) and dissolved in TE buffer (10 mM Tris-HCl, 1 mM EDTA; pH 8.0).

The entire nucleotide sequences of ITS and IGS regions were amplified using polymerase chain reaction (PCR) at 94°C for 45 s, 54°C for 45 s, and 72°C for 90 s (for ITS) or 135 s (for IGS), using the primer sets NS7-NL4 (for ITS) and IGS1-IGS4 (for IGS) (Supplementary Table 3). PCR products were purified using an Illustra ExoStar clean-up kit (GE Healthcare, Buckinghamshire, UK). Dye terminator cycle sequencing reactions were performed using an ABI Big Dye Terminator Cycle Sequencing Ready Reaction Kit (Applied Biosystems, Foster, CA, USA), and reaction products were analyzed using an ABI 3130xl genetic analyzer (Applied Biosystems). The primers used for the PCR and sequencing analyses are listed in Supplementary Table 3.

To detect fine-scale genetic variation of the symbiotic yeasts within each host individual, IGS PCR products from all isolates (i,e., eight colonies per host female) were first sequenced with the primer IGS1. This short sequence (~700 bp) covered the entire IGS1 region, which was subsequently cut out from the raw sequence data. We used this entire IGS1 sequence (namely, the IGS1 haplotype) as a discrimination marker of *Scheffersomyces* symbiotic yeast strains on the grounds that IGS1 exhibits the highest genetic variation among IGS regions (Tanahashi et al., 2017). When more than one IGS1 haplotype was detected from a single host female, we choose one representative isolate for each different IGS1 haplotype for further phylogenetic analyses, following the optimum analytical strategy proposed by Tanahashi et al. (2017). Subsequently, the whole IGS sequence (approximately 2.2 kb) was determined for each representative isolate for each host female (in total 15 sequences) (Supplementary Table 2) using the sequencing primers IGS1, IGS2, IGS3, IGS4, IGS7i and IGS8i.

Although IGS regions are suitable for detecting strain-level genetic differences, they are not commonly used for species identification or phylogenetic analysis at higher taxonomic levels due to high levels of genetic variation as well as insufficient taxonomic coverage in the database. Therefore, for the purposes of species identification and phylogenetic analysis of symbiotic yeasts, we determined 15 ITS sequences from 56 symbiotic yeast isolates from five *Prismognathus dauricus* females and two *Pr. angularis* females (Supplementary Table 2). The ITS PCR products of the representative yeast isolates were sequenced with the primers ITS5 and NL4 (Supplementary Table 3), which were approximately 1.1 kb in size and usually contained whole ITS regions (ITS1, *5.8S rRNA,* and ITS2) of the *Scheffersomyces* yeasts.
