# Supplementary material for: Lateral Transmission of Yeast Symbionts Among Lucanid Beetle Taxa
Source: Front Microbiol. 2021 Dec 14;12:794904. doi: 10.3389/fmicb.2021.794904 (PMC8712881; doi:10.3389/fmicb.2021.794904)
Supplement: Supplementary file 11 [file Data_Sheet_11.PDF]

Supplementary Appendix 3.

Protocol of determining DNA sequences of insect hosts

Genomic DNA was extracted from the muscle tissues of adult *Prismognathus* beetles in ethanol using DNeasy Blood and Tissue Kits (QIAGEN, Hilden, Germany). The *COI* gene (primers C1-J-2183 and L2-N-3014, Simon et al., 1994) was amplified by PCR at 94°C for 3 min, followed by 30 cycles of 94°C for 1 min, 48°C for 1 min, and 72°C for 1 min, with a final 7-min extension at 72°C. The PCR products were purified using the Illustra ExoStar clean-up kit (GE Healthcare, Buckinghamshire, UK). The Dye terminator cycle sequencing reactions were performed using an ABI Big Dye Terminator Cycle Sequencing Ready Reaction Kit (Applied Biosystems, Foster, CA, USA), and electrophoresed using an ABI 3130xl genetic analyzer (Applied Biosystems). The primers used for the PCR and sequencing analyses are listed in Supplementary Table 3. For the sequencing analysis, the same PCR primer sets were used.
